# The emerging importance of autoimmune hepatitis in China

**DOI:** 10.1186/1479-5876-10-S2-A25

**Published:** 2012-10-17

**Authors:** Xiong Ma

**Affiliations:** 1 Renji Hospital, Shanghai Jiao Tong University School of Medicine, Shanghai Institute of Digestive Disease, Shanghai, China

## 

Autoimmune hepatitis (AIH) is a chronic inflammation of the liver caused by an abnormal autoimmune reaction against hepatocytes. The pathogenesis of AIH involves a loss of tolerance to hepatic self antigens in a susceptible host. The diagnosis is based on histological abnormalities, and characteristic clinical and biochemical findings, which include abnormal levels of serum globulins, and the presence of one or more characteristic autoantibodies such as ANA, SMA, and anti-SLA. Interface hepatitis with abundant plasma cells in the infiltrate is characteristic histological features of AIH. Although the prevalence data are scare, AIH has emerging as major course of non-viral chronic hepatitis in China. More and more liver centers launch liver biopsy and serum autoantibody detection regularly. Alertness and awareness of hepatologists and histologic physicians on histologic features of AIH contributes to more and more AIH patients diagnosed in our clinics who were considered as cryptogenic chronic hepatitis previously. The simplified criteria have high sensitivity and specificity for diagnosis of AIH in Chinese patients. The revised original criteria have the complementary role to avoid the false negative diagnosis in atypical AIH patients. Immunosuppressive treatment can attenuate hepatic inflammation, revert fibrosis, and eventually improve the patient’s prognosis and life quality. Corticosteroids, either alone or in combination with azathioprine, are the standard treatment of choice for AIH. Most Chinese patients with AIH show a good response to immunosuppressive treatment, although patients with late-stage or severe disease are less likely to achieve remission. Liver transplantation is the last choice for AIH patients with decompensated end-stage disease. Transfer of regulatory immune cells such as regulatory T cells may become a potential therapy in the near future.

